# Knowledge-based analyses reveal new candidate genes associated with risk of hepatitis B virus related hepatocellular carcinoma

**DOI:** 10.1186/s12885-020-06842-0

**Published:** 2020-05-11

**Authors:** Deke Jiang, Jiaen Deng, Changzheng Dong, Xiaopin Ma, Qianyi Xiao, Bin Zhou, Chou Yang, Lin Wei, Carly Conran, S. Lilly Zheng, Irene Oi-lin Ng, Long Yu, Jianfeng Xu, Pak C. Sham, Xiaolong Qi, Jinlin Hou, Yuan Ji, Guangwen Cao, Miaoxin Li

**Affiliations:** 1 State Key Laboratory of Organ Failure Research, Guangdong Key Laboratory of Viral Hepatitis Research, Institutes of Liver Diseases Research of Guangdong Province, Department of Infectious Diseases and Hepatology Unit, Nanfang Hospital, Southern Medical University, Guangzhou, China; 2 Department of Psychiatry, the University of Hong Kong, Pokfulam, Hong Kong; 3 Ningbo University School of Medicine, Ningbo, China; 4 State Key Laboratory of Genetic Engineering, Collaborative Innovation Center for Genetics and Development, School of Life Sciences, Fudan University, Shanghai, China; 5 Center for Genomic Translational Medicine and Prevention, School of Public Health, Fudan University, Shanghai, China; 6 Program of Computational Genomics & Medicine, NorthShore University HealthSystem, Evanston, IL USA; 7 Department of Public Health Sciences, University of Chicago, Chicago, IL USA; 8 Program for Personalized Cancer Care, NorthShore University HealthSystem, Pritzker School of Medicine, University of Chicago, Evanston, IL USA; 9 Department of Pathology, the University of Hong Kong, Pokfulam, Hong Kong; 10 The Centre for Genomic Sciences, the University of Hong Kong, Pokfulam, Hong Kong; 11 Department of Epidemiology, Second Military Medical University, Shanghai, China; 12 State Key Laboratory for Cognitive and Brain Sciences, the University of Hong Kong, Pokfulam, Hong Kong; 13 State Key Laboratory of Liver Research, the University of Hong Kong, Pokfulam, Hong Kong; 14 Zhongshan School of Medicine, Sun Yat-sen University, Guangzhou, China; 15 Key Laboratory of Tropical Disease Control (SYSU), Ministry of Education, Guangzhou, China

**Keywords:** Knowledge-based genetic association, Susceptibility, Hepatitis B virus, Hepatocellular carcinoma

## Abstract

**Background:**

Recent genome-wide association studies (GWASs) have suggested several susceptibility loci of hepatitis B virus (HBV)-related hepatocellular carcinoma (HCC) by statistical analysis at individual single-nucleotide polymorphisms (SNPs). However, these loci only explain a small fraction of HBV-related HCC heritability. In the present study, we aimed to identify additional susceptibility loci of HBV-related HCC using advanced knowledge-based analysis.

**Methods:**

We performed knowledge-based analysis (including gene- and gene-set-based association tests) on variant-level association *p*-values from two existing GWASs of HBV-related HCC. Five different types of gene-sets were collected for the association analysis. A number of SNPs within the gene prioritized by the knowledge-based association tests were selected to replicate genetic associations in an independent sample of 965 cases and 923 controls.

**Results:**

The gene-based association analysis detected four genes significantly or suggestively associated with HBV-related HCC risk: *SLC39A8*, *GOLGA8M*, *SMIM31*, and *WHAMMP2*. The gene-set-based association analysis prioritized two promising gene sets for HCC, cell cycle G1/S transition and NOTCH1 intracellular domain regulates transcription. Within the gene sets, three promising candidate genes (*CDC45*, *NCOR1* and *KAT2A*) were further prioritized for HCC. Among genes of liver-specific expression, multiple genes previously implicated in HCC were also highlighted. However, probably due to small sample size, none of the genes prioritized by the knowledge-based association analyses were successfully replicated by variant-level association test in the independent sample.

**Conclusions:**

This comprehensive knowledge-based association mining study suggested several promising genes and gene-sets associated with HBV-related HCC risks, which would facilitate follow-up functional studies on the pathogenic mechanism of HCC.

## Background

Hepatocellular carcinoma (HCC) is one of the most common cancers worldwide. With 750,000 new HCC cases diagnosed each year, it is the third leading cause of cancer mortality [1]. As many as 30% of patients diagnosed with hepatitis, fibrosis or cirrhosis ultimately develop HCC. In high endemic areas such as Africa and Asia, at least 60% of HCC is associated with hepatitis B virus (HBV) [2]. However, only a minority of HBV carriers develops HCC. HBV carriers with a family history of HCC were estimated to have over two-fold risk for HCC compared with those without a family history of HCC [3]. Furthermore, genetic complex segregation analysis suggested that major genes may be involved in the genetic predisposition to develop HCC at an earlier age [4].

Genome-wide association study (GWAS) is a widely used strategy for identifying risk loci of complex diseases. Recently, several GWASs on risk of HBV-related HCC were conducted using single-nucleotide polymorphisms (SNPs)-based statistical association tests. Multiple susceptibility loci were identified, including rs17401966 in intron 24 of *KIF1B* at 1p36.22, rs7574865 in intron 3 of *STAT4* at 2q32.2–32.3, rs9275319 between *HLA-DQB1* and *HLA-DQA2* at 6p21.3, rs9272105 between *HLA-DQA1* and *HLA-DRB1* at 6p21.3, and rs455804 in intron 1 of *GRIK1* at 21q21.3 [5–7]. However, these susceptibility loci account for only a small fraction of the contribution of genetics to HBV-related HCC. Identifying additional genetic alterations associated with HBV-related HCC may be difficult due to the relatively weak effects of many individual risk SNPs, which may be unidentifiable with the currently available but relatively small sample sizes [8]. SNP-based statistical association tests alone in GWAS do not have enough power to discover most risk loci for human complex diseases. Gene- and biological pathway-based association analysis has been proposed to enhance statistical power compared with conventional statistical tests, as the former can relieve multiple testing and enrich signals [9]. Moreover, gene- and biological pathway-based analysis also lends itself to introducing more disease-specific knowledge into the analysis.

In the present study, we performed a series of knowledge-based analyses (including gene- and gene-set-based association tests) on variant-level association *p*-values from two in-house GWASs of HBV-related HCC. SNPs within genes prioritized by the knowledge-based analyses were selected for replication in two independent HBV-related HCC case/control samples.

## Methods

### Two existing GWASs on HBV-related HCC

The association *p*-values were obtained from two previous GWASs on HBV-related HCC in Chinese populations for meta-analysis and knowledge-based association analysis. One study [7] contained 2689 chronic HBV carriers (1212 HBV-related HCC cases and 1477 controls) recruited from May 2006 to December 2012 by the Qidong Liver Cancer Institute in Jiangsu Province of Mainland China. The other study [10] consisted of 95 HBV-infected HCC patients (cases) and 97 HBV-infected patients without HCC (controls) recruited at Queen Mary Hospital, Hong Kong. The sample inclusion and exclusion criteria were described in the original papers [7, 10].

### Subjects in replication studies

The subjects in replication, including 965 chronic HBV carriers with HCC as cases and 923 chronic HBV carriers without HCC as controls, were recruited from the affiliated hospitals of the Second Military Medical University, Shanghai, China. All the samples are of Han Chinese descent and have participated in previously published studies [7, 11]. The inclusion and exclusion criteria for all the subjects have been previously described [7, 11]. Briefly, all the subjects were negative for antibodies to hepatitis C virus, or human immunodeficiency virus; and had no other types of liver disease, such as autoimmune hepatitis, toxic hepatitis, and primary biliary cirrhosis. All the controls were chronic HBV carriers and had, by self-report, no history of HCC or other cancers. Chronic HBV carriers were defined as positive for both hepatitis B surface antigen and antibody immunoglobulin G to hepatitis B core antigen for at least 6 months. All the cases were chronic HBV carriers and diagnosed as HCC patients. The diagnosis of HCC was based on a) positive findings on cytological or pathological examination and/or b) positive images on angiogram, ultrasonography, computed tomography and/or magnetic resonance imaging, combined with an Alpha-fetoprotein level ≥ 400 ng/ml. All the cases were confirmed to not have other cancers by an initial screening. The mean (standard deviation) ages of the cases and controls were 50.8 (±12.2) years and 52.9 (±11.2) years, respectively. The male to female ratio were 5.3 in cases and 1.6 in controls, respectively.

The study was performed in accordance with guidelines approved by the local ethical committees from all participating centers involved in both the GWAS stage and the replication stage. A written informed consent to participate in the study was obtained from each subject in accordance with the declaration of Helsinki principles. All study participants approved the storage of their frozen DNA specimens, for research purposes, in our laboratory.

### Genotyping and quality control in replication

Genomic DNA from the peripheral blood of all participants in replication was extracted using the QIAamp DNA Blood Mini Kit (QIAGEN GmbH, Hilden, Germany). Genotyping analyses for replication samples were conducted using the Sequenom MassArray system (Sequenom) according to the manufacturer’s instructions. Genotyping quality was examined by a detailed QC procedure consisting of a 95% successful call rate, duplicate calling of genotypes, and internal positive control samples and two water samples (PCR negative controls) included in each 96-well plate. Genotype analysis was performed by technicians in a blind fashion.

### Meta-analysis of variants

The association *p*-values of untyped SNPs were imputed directly by the tool FAPI (http://grass.cgs.hku.hk/limx/fapi/) [12] with default settings. The *p*-values of the two GWASs were then combined by Stouffer’s Z-score method for meta-analysis on FAPI as well:

$$ {Z}_{meta}=\frac{\sum_{i=1}^N\left({w}_i\ast {z}_i\right)}{\sqrt{\sum_{i=1}^N{w}^2}} $$ where $$ {w}_i=\sqrt{n_i} $$

in which *N* is the number of GWASs, z_i_ is the individual z-score of the i_th_ GWAS study, and n_i_ is the sample size of the i_th_ study.

### Gene-based and gene-set-based analysis

The knowledge-based secondary analysis platform KGG Version 4.0 (http://grass.cgs.hku.hk/limx/kgg/) was used to map the SNPs onto reference genes (UCSC RefGene hg19), and to perform gene-based and gene-set-based association analysis with default settings. Two types of gene-based association tests, GATES [13] and ECS [14], were employed for the analysis which combined SNP-level association signal according to the best significance and accumulated significance respectively. In addition, LDRT [15] was adopted for gene-set-based association analysis. The phased genotypes of Eastern Asian samples in the 1000 Genomes Project [16] were used to account for linkage disequilibrium of SNPs through KGG. The Benjamini-Hochberg approach was used to control false discovery rate (FDR) of genome-wide genes or genes within gene-sets, which is a more powerful multiple testing approach than Bonferroni correction when there are multiple susceptibility genes.

### Variants functional annotation

The genomic annotation tools, HaploReg v4.1 (http://www.broadinstitute.org/mammals/haploreg/haploreg.php) [17] and RegulomeDB Version 1.1 (http://regulomedb.org/) [18], were used to annotate SNPs with epigenomic markers and potential regulatory elements, including regions of DNase I hypersensitivity, binding sites for transcription factors (TFs), promoter regions that have been biochemically characterized to regulate transcription, chromatin states as well as DNase foot printing, PWMs, and DNA Methylation. KGGSeq (Version 1.0) [19, 20] was used to annotate selected SNP with four regulatory or functional prediction scores (including CADD.CScore [21], SuRFR [22], FunSeq2 [23] and cepip [24]).

## Results

**Fig. 1:**
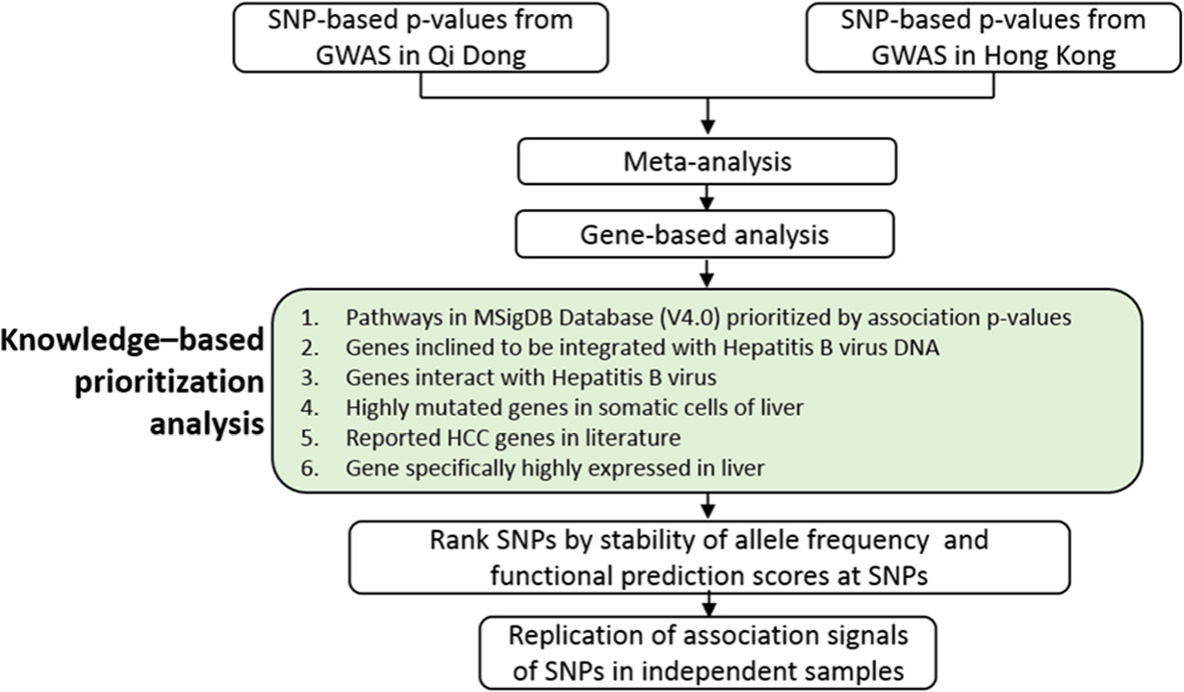
Knowledge-based prioritization framework of SNPs’ statistical *p*-values for association with HCC

We first combined the association *p*-values of variants by meta-analysis from two independent GWASs. Association analyses at genes and multiple functional gene-sets were carried to prioritize potential HBV-related HCC susceptibility genes. A series of prioritized variants were selected from the knowledge-based association analyses to replicate their genetic associations in a group of independent case-control samples. The overall workflow is shown in Fig. 1.

### Genome-wide meta-analysis of two HBV-related HCC GWASs in Chinese populations

Association *p*-values were imputed based on the linkage disequilibrium (LD) pattern in the Eastern Asian Panel from the 1000 Genomes Project. A genome-wide meta-analysis was then performed with SNP *p*-values from two existing Chinese HCC GWASs using the tool FAPI [12]. After quality control (QC), 5,375,073 meta-analysis *p*-values of SNPs were obtained. The Manhattan plot and QQ plots of *p*-values are shown in Supplementary Figure 1 and Supplementary Figure 2, respectively. At the upper tail of the QQ plot, there is a deviation from the 95% confidence level of the non-hypothesis line, suggesting the existence of association signals at some SNPs. The small proportion of significant signals was consistent with the estimated low heritability in the samples by GCTA, 0.063 (±0.028) on the underlying liability scale [25].

### Gene-based association analysis

**Fig. 2:**
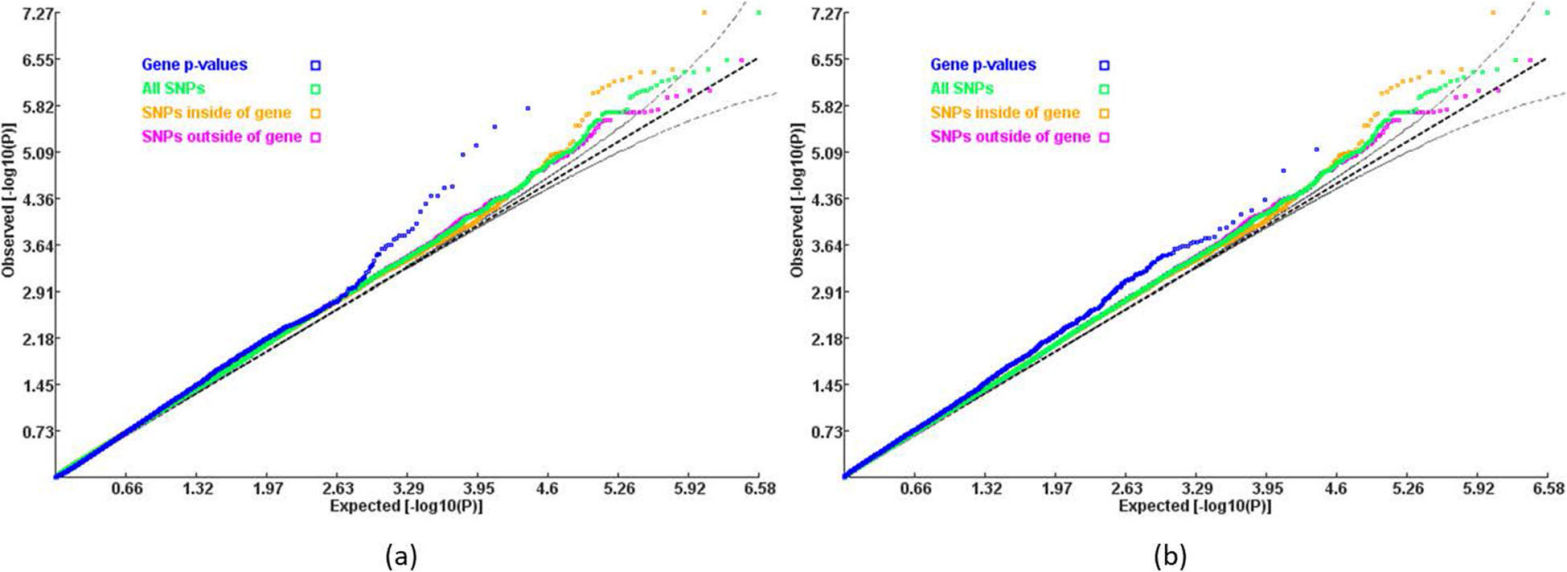
Quantile-quantile plot of gene-based *p*-values and SNP-based *p*-values a) the *p*-values produced by GATES b) the *p*-values produced by ECS

We then used the meta-analysis *p*-values for gene-based association analysis by GATES [13] and ECS [14] on KGG (version 4.0) [26]. In addition to SNPs within the untranslated regions, introns and exons, the meta-analysis *p*-values of SNPs within 5 kb upstream and downstream of a gene were also included in the gene-based association test by GATES and ECS. SNPs in overlapping regions of multiple genes were assigned to all involved genes. The QQ plots of gene-based *p*-values are shown in Fig. 2.

According to the gene-based *p*-values by GATES, two genes, *SLC39A8* and *GOLGA8M* passed the multiple-testing correction by FDR, 0.05 (Table 1). In addition, two genes, *SMIM31* and *WHAMMP2*, had nearly significant *q*-values (< 0.06 by GATES) on the genome (Table 1). Interestingly, *SMIM31*, encoding small integral membrane protein 31*,* was annotated as a long noncoding RNA gene (*LINC01207*) previously. We further annotated the pseudogene, *WHAMMP2*, with known regulatory elements and epigenomic markers by the UCSC genome browser (http://genome.ucsc.edu). Although it is annotated as a pseudogene, there are multiple regulatory factors binding sites and epigenomic markers in *WHAMMP2* (See Supplementary Figure 3). These annotations imply that this gene is also functionally active despite its non-protein-coding function. The other gene-based test, ECS, detected no significant gene. The gene with smallest *p*-value (7.5E-06) is *RNF157-AS1*.

**Table 1:** The top 5 genes according to gene-based *p*-values by GATES and ECS, respectively

Gene	CHR	Type	#SNP	GATES		ECS	
				Nominal ***p***	Corrected ***p***^**a**^	Nominal ***p***	Corrected ***p***^**a**^
***SLC39A8***	4	protein-coding gene	222	1.63E-06	0.04138	0.22495	0.83656
***GOLGA8M***	15	protein-coding gene	11	3.19E-06	0.04138	1.57E-05	0.20422
***SMIM31***	4	protein-coding gene	300	6.43E-06	0.05560	0.00424	0.52238
***WHAMMP2***	15	pseudogene	14	9.03E-06	0.05858	0.00031	0.32182
***CLDN5***	22	protein-coding gene	22	2.76E-05	0.12596	0.01665	0.62074
***RNF157-AS1***	17	non-coding RNA	20	3.84E-05	0.12655	7.50E-06	0.19448
***LRRC9***	14	other	348	0.00249	0.62765	4.46E-05	0.32182
***LINC02062***	5	non-coding RNA	150	0.02402	0.69927	0.00007	0.32182
***TTL***	2	protein-coding gene	101	0.01254	0.65610	7.64E-05	0.32182

Note. CHR: chromosome

^a^ The *p*-values are corrected by the Benjamini-Hochberg FDR approach. *SLC39A8*, *GOLGA8M*, *SMIM31*, *WHAMMP2* and *CLDN5* are the top five genes according to GATES. *RNF157-AS1*, *GOLGA8M*, *LRRC9*, *LINC02062* and *TTL* are the top five genes according to ECS

### Prioritization of genes in different gene-sets

To select more promising candidate genes for replication in independent samples, we resorted to a series of gene-set resources to prioritize genes with suggestive association *p*-values. We first examined the association with HCC in 1057 canonical pathways curated in the Molecular Signatures Database (MSigDB V 4.0), after removing the pathways containing too few (< 5) or too many (> 300) genes. The gene-set-based association *p*-value was performed by LDRT [15] on KGG. Although no gene-sets passed multiple testing (FDR *q* < 0.05), several promising functional gene sets are prioritized. The top two gene sets according to the *p*-value are the cell cycle G1/S transition (*p* = 5.5E-4) and the NOTCH1 intracellular domain regulates transcription (*p* = 7.1E-4). In the G1/S transition gene set, 12 out 99 genes had gene-based association (*p* < 0.05, See details in Supplementary Excel Table 1). The gene with the smallest *p*-value is *CDC45* (*p* = 1.1E-4) in this gene set. In the gene set of NOTCH1 intracellular domain regulates transcription, 10 out 40 genes had gene-based association (*p* < 0.05, See details in Supplementary Excel Table 1). In the set, *NCOR1* had the smallest *p*-value (*p* = 5.8E-3). The second gene, *KAT2A*, had similar *p*-value (6.6E-3).

Then, we investigated whether the genes highly and specifically expressed in human liver were associated with HCC. In the database, Tissue-specific Gene Expression and Regulation (TiGER, http://bioinfo.wilmer.jhu.edu/tiger/), 309 genes preferentially expressed in liver were retrieved. In the human proteome atlas (http://www.proteinatlas.org/humanproteome), 433 genes showing elevated expression of proteins in liver compared to other tissue types were retrieved as well. To reduce potential false positives, we only used overlapping genes in the two sets. As a result, a total of 189 genes were obtained. Three genes (*PAH*, *UGT2B10* and *UROC1*) had the FDR *q* values < 0.1 by ECS while GATES did not detect any significant gene (See the genes and *p*-values in Table 2 and Supplementary Table 1).

**Table 2:** Genetic association *p*-values of genes preferentially expressed in liver

Gene Symbol^a^	GATES *p*	ECS *p*	ECS *q*	CHR	Start Position	Length (BP)	Number of SNPs
*PAH*	> 0.05	0.00035	0.064	12	103,230,666	80,356	266
*UGT2B10*	0.01504	0.00079	0.073	4	69,870,294	172,553	122
*UROC1*	0.02728	0.00138	0.085	3	126,200,008	36,608	92
*TF*	0.00293	0.01388	0.386	3	133,465,236	50,249	288
*C4A*	> 0.05	0.01472	0.386	6	31,949,833	20,624	20
*SLCO1B1*	> 0.05	0.01528	0.386	12	21,284,127	108,603	298
*C5*	> 0.05	0.01605	0.386	9	123,761,950	50,603	150
*GSTA2*	0.04013	0.01864	0.386	6	52,614,884	13,389	59
*C4B*	> 0.05	0.02012	0.386	6	31,982,571	12,113	38
*HAO1*	0.03276	0.02195	0.386	20	7,863,631	57,474	118
*NAT2*	0.01023	0.02308	0.386	8	18,248,791	9934	70
*GSTA1*	0.01104	> 0.05	0.697	6	52,656,170	12,444	50
*AQP9*	0.03199	> 0.05	0.733	15	58,430,579	47,531	182
*SAA2*	0.04169	> 0.05	0.804	11	18,266,786	3429	55
*APOA2*	0.04277	> 0.05	0.733	1	161,192,081	1337	22

Note. CHR: chromosome; *BP* base pairs

^a^ Only the genes with a *p*-value less than 0.05 are listed in this table. The whole gene list is shown in Supplementary Table 1

We also examined the association of recurrent integrated genes by HBV reported in previous studies [27–30], the genes reported to be genetically associated with HBV-related HCC risk in previous studies, and HCC risk genes defined by COSMIC database (http://cancer.sanger.ac.uk/cosmic). However, none of the genes had a promising association *p*-value with HCC in our samples (see the genes and *p*-values in Supplementary Tables 2, 3 and 4).

### Replication study in independent samples

We replicated genetic association at genes prioritized by the above gene-based and gene-set-based associations in a group of independent HBV-related HCC case-control samples. Due to budget limit, only 21 SNPs were selected for the replication. The SNPs were at prioritized genes according to consistency of their allele frequencies in ancestry matched reference panel in the 1000 Genomes Project and HapMap Project, and/or their predicted functional importance by RegulomeDB (http://regulomedb.org/) with regulatory elements (See examples in Supplementary Figures 3 and 4). After the genotype quality assessment, two SNPs were excluded because they failed to pass the Hardy-Weinberg equilibrium test (*p* < 0.001).

Three genetic models (additive, dominant and recessive) were considered under a logistic regression framework in which the HCC status was adjusted for sex and age. None of the 19 SNPs survived the multiple Bonferroni correction for family-wise error rate 0.05. Only two SNPs, rs17343667 and rs389883, had a nominal *p*-value below 0.05. The rs17343667, which is located in the first intron of *EIF2AK1*, had an association *p*-value equal to 0.02 under the dominant model with an odds ratio of 1.27 for the minor allele, which was found to have a risk effect in both original Qidong and Hong Kong GWAS samples (Table 3). However, its *p*-value was only 0.15 under the additive model. The rs389883, which is in intron region of *STK19*, had *p*-values of 0.026 and 0.032 for HCC association under additive and recessive models, respectively, with a protective effect at the minor allele G. However, in the original Qidong GWAS sample and Hong Kong GWAS sample, G was estimated to have a risk effect. Therefore, the SNP-level replication was generally negative.

**Table 3:** Summary of genetic association results in the replication

	CHR	SNP	BP	CADD.CScore	SuRFR	FunSeq2	HCCCell_Prob	RegulomeDB	A1	A2	Additive^**a**^		Dominant^**a**^		Recessive^**a**^	
											OR (95% CI)	***p***	OR (95% CI)	***p***	OR (95% CI)	***p***
	1	rs3813948	207,269,858	−0.039	14.356	0.7635	0.796	5	C	T	1.04 (0.87–1.24)	0.668	1.02 (0.83–1.25)	0.845	1.27 (0.72–2.23)	0.412
	2	rs60325402	16,077,873	0.144	17.3	0.1852	0.370	5	A	T	0.85 (0.59–1.21)	0.360	0.83 (0.58–1.19)	0.319	- ^b^	0.999
	3	rs7612684	178,984,575	−0.163	19.334	0.8109	0.370	4	G	A	0.79 (0.59–1.05)	0.105	0.76 (0.56–1.03)	0.080	1.53 (0.23–10.01)	0.657
	3	rs76863563	178,987,536	−0.498	15.493	0.1881	0.370	5	C	T	0.91 (0.66–1.26)	0.567	0.89 (0.64–1.24)	0.506	2.00 (0.17–24.06)	0.587
	5	rs116966235	57,794,613	−0.636	.	0.1852	0.370	3a	G	A	1.07 (0.79–1.46)	0.670	1.06 (0.78–1.45)	0.705	- ^b^	0.999
	5	rs12514619	1,783,655	1.741	7.556	2.705	0.370	2b	C	T	1.10 (0.94–1.28)	0.252	1.06 (0.87–1.28)	0.563	1.45 (0.96–2.20)	0.078
	6	rs389883	31,947,460	0.142	14.213	1.623	0.370	1f	G	T	0.86 (0.75–0.98)	0.026	0.86 (0.71–1.03)	0.108	0.73 (0.55–0.97)	0.032
	6	rs615672	32,574,171	−0.162	4.627	0.7972	0.370	6	G	C	0.93 (0.81–1.07)	0.293	0.98 (0.81–1.17)	0.795	0.74 (0.54–1.01)	0.056
	7	rs17343667	6,065,194	0.392	15.543	0.8898	0.370	1f	A	G	1.11 (0.96–1.27)	0.151	1.27 (1.04–1.55)	0.020	0.97 (0.76–1.24)	0.792
	7	rs55744175	18,332,396	2.275	17.195	0.6909	0.370	5	A	G	1.05 (0.90–1.24)	0.524	1.07 (0.89–1.30)	0.474	1.02 (0.65–1.62)	0.924
	8	rs16898013	124,138,891	0.780	17.314	0	0.370	3a	A	G	0.85 (0.63–1.16)	0.306	0.85 (0.61–1.16)	0.304	0.82 (0.11–6.23)	0.847
	8	rs2275959	37,455,059	0.245	6.377	0.3114	0.863	4	A	G	0.98 (0.86–1.12)	0.791	1.02 (0.83–1.25)	0.854	0.93 (0.74–1.16)	0.503
	8	rs2736020	15,714,529	−0.002	3.977	9.418E-161	0.370	7	C	T	1.09 (0.94–1.25)	0.255	1.13 (0.93–1.36)	0.209	1.06 (0.79–1.44)	0.687
	10	rs3001719	10,409,365	−0.113	3.277	0.1852	0.370	5	G	T	1.08 (0.94–1.25)	0.288	1.11 (0.92–1.34)	0.261	1.08 (0.76–1.52)	0.674
	11	rs10897243	62,043,174	−0.497	15.511	4.535E-33	0.370	6	G	C	0.92 (0.79–1.08)	0.311	0.93 (0.77–1.13)	0.468	0.81 (0.55–1.20)	0.296
	12	rs79475045	39,083,557	−0.264	15.822	0.1881	0.370	5	T	G	0.88 (0.73–1.06)	0.189	0.91 (0.74–1.12)	0.377	0.55 (0.29–1.06)	0.072
	12	rs979722	118,217,304	0.014	15.899	0.4365	0.370	7	C	T	1.05 (0.91–1.20)	0.512	1.05 (0.87–1.27)	0.597	1.09 (0.81–1.46)	0.577
	16	rs12918376	56,558,181	−0.025	12.043	4.562E-74	0.370	6	T	G	1.11 (0.96–1.27)	0.153	1.11 (0.91–1.35)	0.303	1.19 (0.92–1.54)	0.182
	20	rs2425046	33,871,661	0.090	17.787	1.78	0.918	2b	C	T	0.98 (0.77–1.24)	0.848	0.92 (0.72–1.19)	0.540	2.20 (0.79–6.14)	0.134

Note. *CHR* chromosome, *BP* base pairs, *OR* odd ratio, *CI* confidence interval, *A1* minor allele, *A2* major allele, CADD.CScore, SuRFR and FunSeq2 scores are annotated by KGGSeq (V1.0). HCCCell_Prob: Probability of cell type-specific regulation in GENCODE liver cancer cells (HepG2)

^a^ This model was tested under Logistic regression model with adjustment for age and sex

^b^ The value is not available

## Discussion

This study utilized knowledge-based approaches to mine new susceptibility loci of HBV-related HCC in existing HBV-related HCC GWAS data sets. The gene-based association analysis suggested four suggestively significant genes including *SLC39A8*, *GOLGA8M*, *SMIM31*and *WHAMMP2*. The gene-set-based association analysis prioritized three top genes (*CDC45*, *NCOR1* and *KAT2A*), which have been implicated with HCC previously, mainly through regulated expression. In addition, three genes, *PAH*, *UGT2B10* and *UROC1* were also highlighted when multiple-testing correction (FDR *q* < 0.1) was performed among genes highly and specifically expressed in human liver. However, probably due to small small sample size, no associations prioritized by the knowledge-based association analysis were successfully replicated in an independent sample. The rs17343667 of *EIF2AK1* is the only one with suggestive significance. Furthermore, our analysis also suggested that the germline susceptibility loci of HBV-related HCC are unlikely to be enriched in recurrent targeted genes of HBV infection, or HCC risk genes with many somatic mutations.

According to our estimation, HCC has relatively low heritability (6.3%). It is unlikely that there are susceptibility genes or loci of large effect size. The association test enriched the association signals of multiple loci in multiple genes with low effect size so that the susceptibility pathways and gene sets can be prioritized. Moreover, it is easier to prioritize potential susceptibility genes given the prioritized gene sets. In our analysis, a non-trivial fraction of genes within the gene sets achieved moderately significant *p*-values. It is likely that some of the genes may achieve genome-wide significance when sample sizes are increased. However, almost all of the genes would be ignored by the widely-adopted genome-wide *p*-value threshold (5E-8) in the present samples (1307 cases vs.1574 controls).

Our study is the first to show that genetic variations of two genes (*SLC39A8* and *GOLGA8M*) are significantly associated with the development of HBV-related HCC. *SLC39A8* encodes a member of the *SLC39* family of solute-carrier genes (Zrt/Irt-like protein 8, ZIP8), which may play an important role in autophagy during ethanol exposure in human hepatoma cells [31]. Liu et al. suggested that hepatic ZIP8 deficiency was associated with tumor formation [32]. Moreover, *SLC39A8* has been reported to regulate IFN-γ level in T cells [33] and influence trace element homeostasis in liver [34, 35], which may be relevant to the development of HCC. *GOLGA8M* encodes golgin A8 family member M. Although it has not been linked to cancer, a study suggested that palindromic *GOLGA8* core duplicons promoted chromosome microdeletion and evolutionary instability [36]. In addition, two other genes (*SMIM31* and *WHAMMP2*) also achieved suggestively significant *p*-values. *SMIM31* has been implicated as a biomarker for survival of colorectal adenocarcinoma [37] and promoting proliferation of lung adenocarcinoma [38]. *RNF157-AS1*, which was implicated by ECS, is an antisense RNA gene. Differential expression between tumor and non-tumor tissue at this gene has been founded in lung cancer [39] and ovarian cancer [40]. Anyhow, functional validation studies are needed to explore the mechanisms of the potential roles of these genes in risk of HBV-related HCC.

The successful prioritization of two gene sets that are highly relevant to cancer development also implies the power of the knowledge-based analysis. The top two functional gene-sets are cell cycle G1/S transition and NOTCH1 intracellular domain regulates transcription. There have been numerous studies linking these functional gene sets to HCC [41–44]. For example, Wang et al. recently showed that lnc-UCID promotes G1/S Transition and hepatoma growth by preventing DHX9-Mediated CDK6 down-regulation [41]. As the gene with the smallest *p*-value in the cell cycle G1/S transition gene set, *CDC45* encodes cell division control protein 45 and has been linked to many cancers according to its expression, including HCC [45] and colorectal cancer [46]. *NCOR1*, the gene with the smallest *p*-value in the gene set of NOTCH1 intracellular domain regulates transcription, encodes a protein that mediates ligand-independent transcription repression of thyroid-hormone and retinoic-acid receptors, which may regulate de novo fatty acids synthesis in liver regeneration and hepatocarcinogenesis in mice [47]. For another gene with similar *p*-value as *NCOR1* in the gene set of NOTCH1 intracellular domain regulates transcription, *KAT2A* encodes lysine acetyltransferase 2A and was linked to HCC. For instance, Majaz et al. suggested that *KAT2A* may promote human HCC progression by enhancing AIB1 expression [48]. The highly and specifically expression in human liver is also an effective stratum for prioritization of HCC susceptibility genes. When multiple testing correction is carried out in this gene set, three genes *PAH*, *UGT2B10* and *UROC1* achieved suggestive significance level (FDR *q* < 0.1). All of the three genes have been implicated with HCC by multiple studies. The most significant gene *PAH* (*p* = 3.5E-4 and *q* = 0.064) has the largest number of literature supports, that is, many studies have implicated this gene in development of HCC. For example, Miller et al. showed p-Chlorphenylalanine effect on phenylalanine hydroxylase in hepatoma cells in culture [49]. Gopalakrishnan and Anderson showed the epigenetic activation of phenylalanine hydroxylase in mouse erythroleukemia cells by the cytoplast of rat hepatoma cells [50]. *UGT2B10* (*p* = 7.9E-4) encodes UDP-Glucuronosyltransferase 2B10. Hanioka et al. showed that expression of *UGT2B* isoforms (including *UGT2B10*) was significantly increased by AFB1 in HepG2 cells [51]. *UROC1* (*p* = 1.4E-3) encodes enzyme involved in histidine catabolism, metabolizing urocanic acid to formiminoglutamic acid. Zhang et al. showed that *UROC1* may play important roles in HCC development, especially alcohol-related HCC development and progression [52].

The negative findings in the curated gene sets of recurrent targeted genes of HBV infection and HCC risk genes with many somatic mutations are unexpected to some extent. Both gene sets appeared to be biologically relevant to the development of HCC. In the analyses, there were no trends that genes with smaller HCC association *p*-values were enriched in the gene sets. These results suggest that the biological context or connection of underlying susceptibility genes is elusive, and that it is difficult to simply use our current knowledge to identify the unknown susceptibility genes of HCC. Using larger sample for hypothesis-free GWASs is likely the only effective way for identification of HCC risk genes at present.

The issue of negative association at variants in replication sample is consistent with that in the discovery sample. Due to small effect size, no variants in the discovery GWAS sample of 1307 HBV-related HCC cases and 1574 controls had a *p*-value less than the widely-adopted genome-wide cutoff (5E-8). It was the gene-based association analysis combing the *p*-values of multiple SNPs that achieved genome-wide significant *p*-values at some genes. Because of budget limit, however, most genes only had one selected SNPs to maximize the total number of genes for replication. Therefore, we were unable to carry out the gene-based association in the replication study as we did in the GWAS sample. Unfortunately, probably due to low effect size, no variants achieved significant *p*-value in the replication sample of 965 HBV-related HCC cases and 923 controls. The SNP-level negative replication implies either more powerful knowledge-based association study or larger sample is needed for identifying HCC susceptibility genes.

## Conclusion

We performed the first systematic gene- and gene-set-based association study of HCC. Our study suggested several promising genes significantly associated with HCC risk, which may shed insights into pathogenic mechanisms of this fatal disorder. However, the failure in replication study also implies small effect size of the susceptibility genes. More hypothesis-free genetic studies with larger sample sizes are needed to elucidate the susceptibility genes and mechanisms of HCC.

## Supplementary information

**Additional file 1: Supplementary Figure 1.** Manhattan plots of *p*-values of 5,375,073 SNPs obtained by a meta-analysis of two HBV-related HCC GWASs in Chinese populations. **Supplementary Figure 2.** The quantile-quantile plots of *p*-values of 5,375,073 SNPs obtained by a meta-analysis of two HBV-related HCC GWASs in Chinese populations. **Supplementary Figure 3.** Regulatory annotations at gene WHAMMP2. **Supplementary Figure 4.** Visualization of regulatory annotation at rs17343667 by RegulomeDB. **Supplementary Table 1.** Genetic association *p*-values of genes preferentially expressed in liver. **Supplementary Table 2.** Association *p*-values of genes frequently intergraded by HBV. **Supplementary Table 3.** Association *p*-values of genes with significant association in previous studies. **Supplementary Table 4.** Association *p*-values of COSMIC HCC risk genes.

**Additional file 2:** Excel table of canonical pathways of MSigDB with *p* < 0.05 by gene-set based association tests.

## Data Availability

Please contact author for data requests.

## Abbreviations

eQTL: Expression quantitative trait locus
FDR: False discovery rate
GWAS: Genome-wide associated studies
HBV: Hepatitis B virus
HCC: Hepatocellular carcinoma
LD: Linkage disequilibrium
QC: Quality control
SLE: Systemic lupus erythematosus
SNP: Single nucleotide polymorphism
TF: Transcription factor
