# Supplementary material for: Knowledge-based analyses reveal new candidate genes associated with risk of hepatitis B virus related hepatocellular carcinoma
Source: BMC Cancer. 2020 May 11;20:403. doi: 10.1186/s12885-020-06842-0 (PMC7216662; doi:10.1186/s12885-020-06842-0)
Supplement: Supplementary file 1 — Additional file 1: Supplementary Figure 1. Manhattan plots of p-values of 5,375,073 SNPs obtained by a meta-analysis of two HBV-related HCC GWASs in Chinese populations. Supplementary Figure 2. The quantile-quantile plots of p-values of 5,375,073 SNPs obtained by a meta-analysis of two HBV-related HCC GWASs in Chinese populations. Supplementary Figure 3. Regulatory annotations at gene WHAMMP2. Supplementary Figure 4. Visualization of regulatory annotation at rs17343667 by RegulomeDB. Supplementary Table 1. Genetic association p-values of genes preferentially expressed in liver. Supplementary Table 2. Association p-values of genes frequently intergraded by HBV. Supplementary Table 3. Association p-values of genes with significant association in previous studies. Supplementary Table 4. Association p-values of COSMIC HCC risk genes. [file 12885_2020_6842_MOESM1_ESM.docx]

**Supplementary Materials**

**List of supplementary materials**

- Supplementary Figure 1. Manhattan plots of *p*-values of 5,375,073 SNPs obtained by a meta-analysis of two HBV-related HCC GWASs in Chinese populations.
- Supplementary Figure 2. The quantile-quantile plots of *p*-values of 5,375,073 SNPs obtained by a meta-analysis of two HBV-related HCC GWASs in Chinese populations.
- Supplementary Figure 3. Regulatory annotations at gene WHAMMP2.
- Supplementary Figure 4. Visualization of regulatory annotation at rs17343667 by RegulomeDB.
- Supplementary Table 1. Genetic association *p*-values of genes preferentially expressed in liver.
- Supplementary Table 2. Association *p*-values of genes frequently intergraded by HBV
- Supplementary Table 3. Association *p*-values of genes with significant association in previous studies
- Supplementary Table 4. Association *p*-values of COSMIC HCC risk genes.


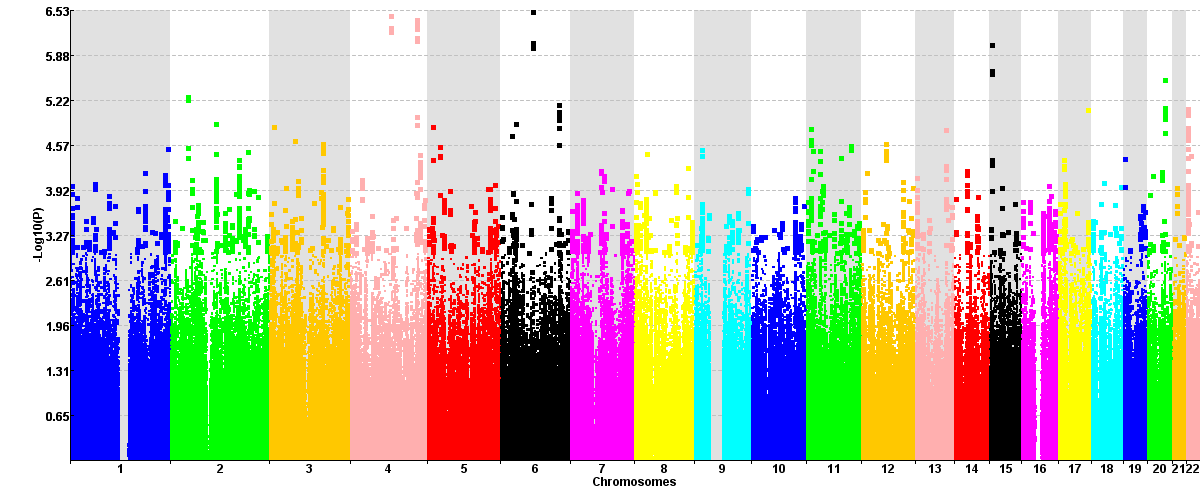


**Supplementary Figure 1**. Manhattan plots of *p*-values of 5,375,073 SNPs obtained by a meta-analysis of two HBV-related HCC GWASs in Chinese populations. The *p*-values are taken –log10 to visualize significance of small *p*-values.


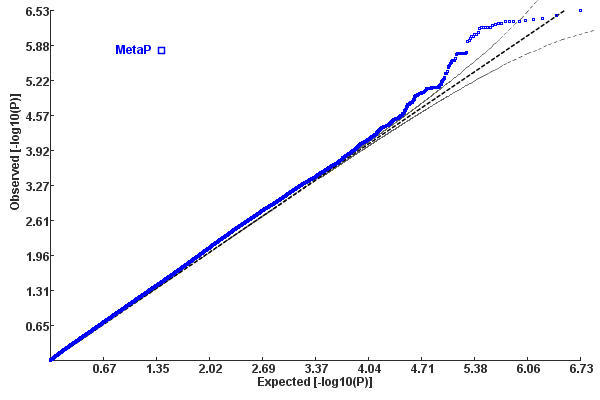


**Supplementary Figure 2**. The quantile-quantile plots of *p*-values of 5,375,073 SNPs obtained by a meta-analysis of two HBV-related HCC GWASs in Chinese populations.


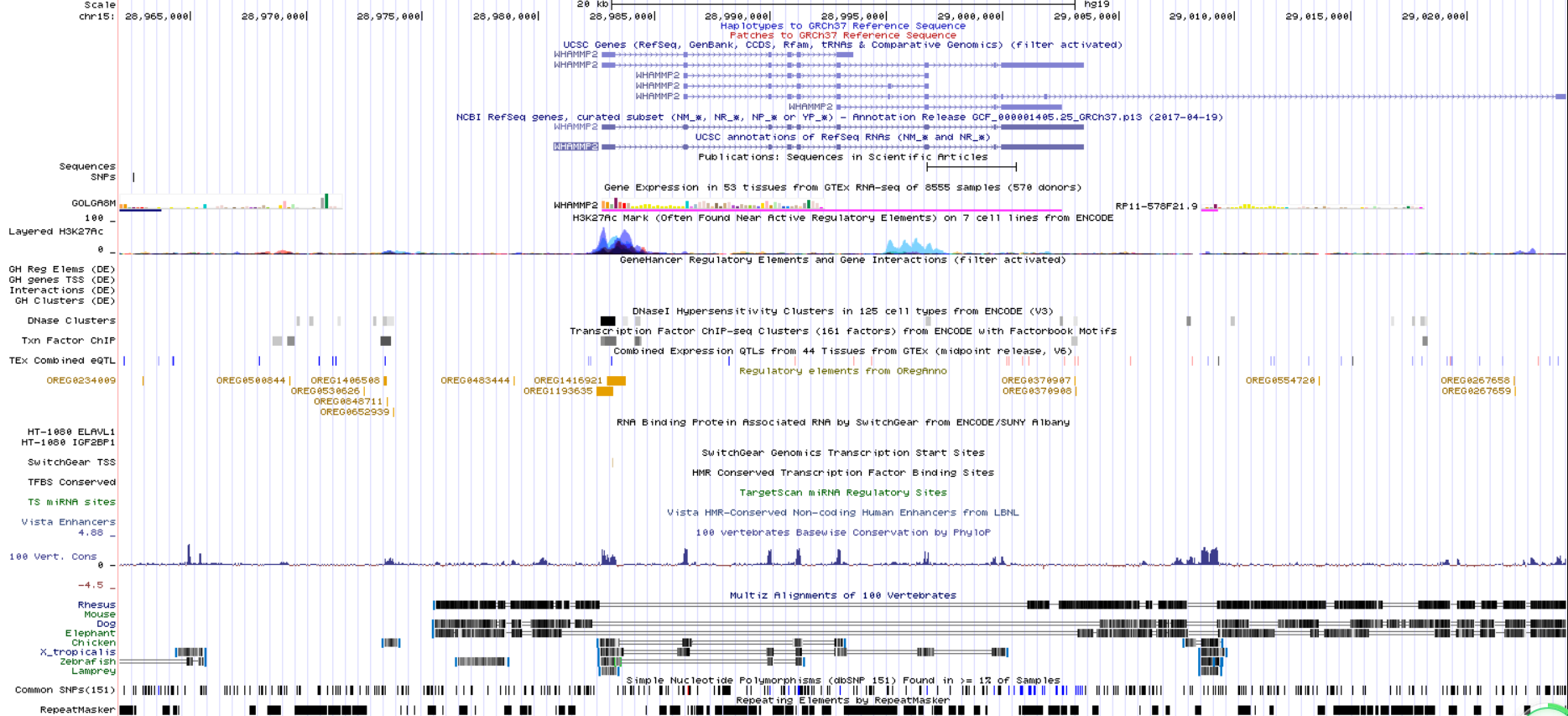


**Supplementary Figure 3**. Regulatory annotations at gene *WHAMMP2*. This plot is produced by UCSC genome browser (<http://genome.ucsc.edu>). The regulatory options (ENCODE Regulation, Chromatin Interactions, DNA Methylation, Histone Modification, Transcription Factor Binding Tracks, Regulatory elements from ORegAnno, CD34 DnaseI, and Vista Enhancers) were turned on.


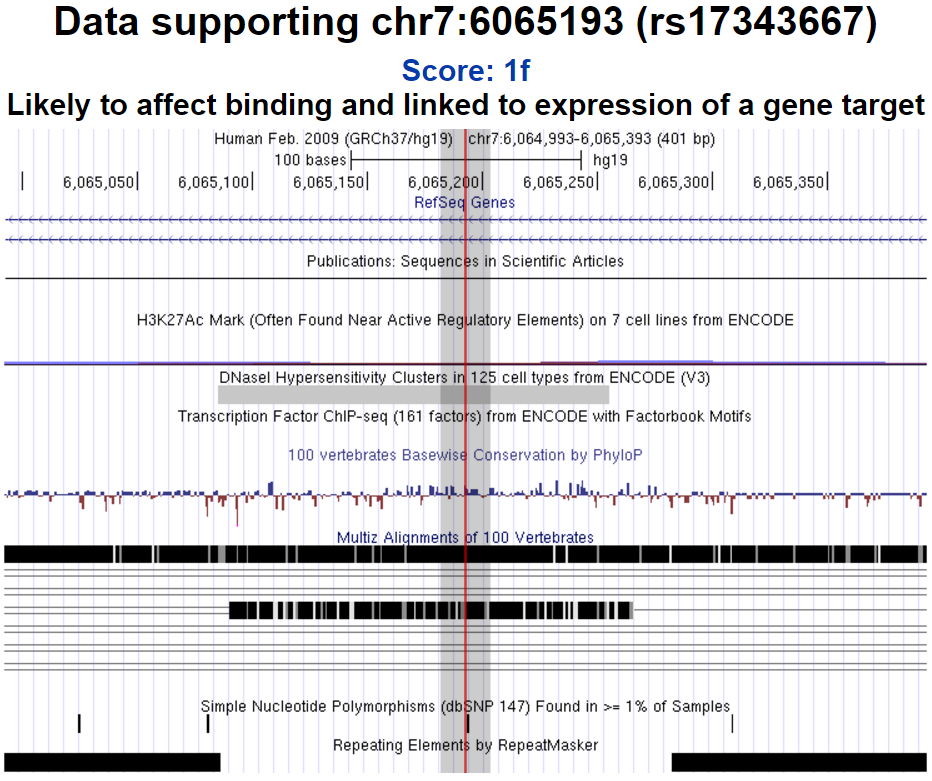


**Supplementary Figure 4**. Visualization of regulatory annotation at rs17343667 by RegulomeDB. This plot is produced by RegulomeDB (<http://www.regulomedb.org>) with RS ID.

**Supplementary Table 1**. Genetic association *p*-values of genes preferentially expressed in liver

| Symbol | GATES-*P* | ECS-*P* | CHR | Start Position | Length (BP) | Number of SNPs |
| --- | --- | --- | --- | --- | --- | --- |
| *PAH* | 0.24765 | 0.00035 | 12 | 103230666 | 80356 | 266 |
| *UGT2B10* | 0.01504 | 0.00079 | 4 | 69870294 | 172553 | 122 |
| *UROC1* | 0.02728 | 0.00138 | 3 | 126200008 | 36608 | 92 |
| *TF* | 0.00293 | 0.01388 | 3 | 133465236 | 50249 | 288 |
| *C4A* | 0.06735 | 0.01472 | 6 | 31949833 | 20624 | 20 |
| *SLCO1B1* | 0.13535 | 0.01528 | 12 | 21284127 | 108603 | 298 |
| *C5* | 0.21972 | 0.01605 | 9 | 123761950 | 50603 | 150 |
| *GSTA2* | 0.04013 | 0.01864 | 6 | 52614884 | 13389 | 59 |
| *C4B* | 0.129 | 0.02012 | 6 | 31982571 | 12113 | 38 |
| *HAO1* | 0.03276 | 0.02195 | 20 | 7863631 | 57474 | 118 |
| *NAT2* | 0.01023 | 0.02308 | 8 | 18248791 | 9934 | 70 |
| *C4BPA* | 0.24822 | 0.0511 | 1 | 207277577 | 40740 | 71 |
| *TAT* | 0.09868 | 0.05446 | 16 | 71599562 | 11433 | 26 |
| *AGXT2* | 0.09673 | 0.05659 | 5 | 34998206 | 49848 | 215 |
| *TDO2* | 0.28695 | 0.06283 | 4 | 156824847 | 16711 | 54 |
| *F2* | 0.18784 | 0.06361 | 11 | 46740762 | 20294 | 28 |
| *SLCO1B3* | 0.05607 | 0.06376 | 12 | 20975481 | 94362 | 536 |
| *LRG1* | 0.10587 | 0.06444 | 19 | 4536413 | 3635 | 6 |
| *APOH* | 0.16442 | 0.06527 | 17 | 64208150 | 17383 | 79 |
| *ASGR1* | 0.07453 | 0.07133 | 17 | 7076750 | 5939 | 6 |
| *C8B* | 0.31258 | 0.08504 | 1 | 57394882 | 36806 | 97 |
| *CYP2A7* | 0.13973 | 0.08514 | 19 | 41381343 | 7314 | 87 |
| *FGL1* | 0.06307 | 0.08788 | 8 | 17721899 | 31014 | 197 |
| *SERPINA3* | 0.28537 | 0.09628 | 14 | 95078727 | 11663 | 39 |
| *FETUB* | 0.32071 | 0.09832 | 3 | 186358187 | 12743 | 48 |
| *GSTA1* | 0.01104 | 0.10232 | 6 | 52656170 | 12444 | 50 |
| *RBP4* | 0.07854 | 0.10538 | 10 | 95351592 | 9400 | 44 |
| *ALDH8A1* | 0.12476 | 0.10623 | 6 | 135238530 | 32709 | 65 |
| *CYP3A7* | 0.14214 | 0.1099 | 7 | 99302658 | 30161 | 56 |
| *CPB2* | 0.12493 | 0.1235 | 13 | 46627321 | 51847 | 156 |
| *FGG* | 0.07183 | 0.13238 | 4 | 155525322 | 8486 | 18 |
| *APOA2* | 0.04277 | 0.13816 | 1 | 161192081 | 1337 | 22 |
| *AMBP* | 0.19443 | 0.14423 | 9 | 116822407 | 18173 | 79 |
| *HRG* | 0.1571 | 0.14658 | 3 | 186383802 | 12221 | 62 |
| *APOM* | 0.28606 | 0.14685 | 6 | 31623670 | 2317 | 28 |
| *HABP2* | 0.42501 | 0.14977 | 10 | 115312811 | 36549 | 169 |
| *INHBC* | 0.23798 | 0.15154 | 12 | 57828566 | 17279 | 39 |
| *AQP9* | 0.03199 | 0.15963 | 15 | 58430579 | 47531 | 182 |
| *SERPINA1* | 0.31716 | 0.1627 | 14 | 94843083 | 11856 | 56 |
| *FMO3* | 0.28652 | 0.16545 | 1 | 171060045 | 26914 | 108 |
| *SERPINC1* | 0.12826 | 0.16577 | 1 | 173872941 | 13524 | 37 |
| *NR1H4* | 0.2477 | 0.16933 | 12 | 100897137 | 61055 | 145 |
| *HSD17B6* | 0.11868 | 0.17135 | 12 | 57157107 | 24467 | 42 |
| *CYP7A1* | 0.26478 | 0.18451 | 8 | 59402736 | 9986 | 42 |
| *DPYS* | 0.51541 | 0.1864 | 8 | 105391658 | 87625 | 171 |
| *C3* | 0.08473 | 0.18662 | 19 | 6677714 | 42947 | 148 |
| *ADH1C* | 0.3069 | 0.18787 | 4 | 100257653 | 16250 | 107 |
| *RIDA* | 0.13179 | 0.2046 | 8 | 99114571 | 14828 | 54 |
| *ASGR2* | 0.30112 | 0.20635 | 17 | 7004640 | 12989 | 120 |
| *EHHADH* | 0.2643 | 0.21175 | 3 | 184908411 | 63426 | 109 |
| *CYP4F2* | 0.16832 | 0.22958 | 19 | 15988832 | 20052 | 102 |
| *ITIH4* | 0.42063 | 0.24111 | 3 | 52847005 | 17683 | 37 |
| *HAO2* | 0.3818 | 0.24778 | 1 | 119911407 | 25346 | 60 |
| *KNG1* | 0.83669 | 0.25032 | 3 | 186435147 | 27052 | 179 |
| *ORM1* | 0.14499 | 0.26023 | 9 | 117085302 | 3457 | 44 |
| *DIO1* | 0.45933 | 0.26235 | 1 | 54359859 | 16900 | 86 |
| *HPR* | 0.09131 | 0.26734 | 16 | 72097124 | 14021 | 29 |
| *FGA* | 0.14675 | 0.27409 | 4 | 155506426 | 5468 | 21 |
| *FMO5* | 0.2928 | 0.28831 | 1 | 146657839 | 39092 | 96 |
| *PON3* | 0.16061 | 0.28901 | 7 | 94989183 | 36504 | 56 |
| *CYP2B6* | 0.51552 | 0.29436 | 19 | 41497186 | 27117 | 101 |
| *ANG* | 0.26455 | 0.30522 | 14 | 21156922 | 5420 | 41 |
| *CXCL2* | 0.36908 | 0.31573 | 4 | 74962756 | 2156 | 10 |
| *SULT2A1* | 0.28813 | 0.33316 | 19 | 48373723 | 15849 | 59 |
| *ALB* | 0.36284 | 0.33414 | 4 | 74270003 | 17196 | 40 |
| *PIPOX* | 0.4244 | 0.33779 | 17 | 27370158 | 14076 | 21 |
| *CPN1* | 0.67109 | 0.33873 | 10 | 101801949 | 39677 | 89 |
| *ALDOB* | 0.27218 | 0.34176 | 9 | 104182841 | 15215 | 33 |
| *SERPINA5* | 0.52521 | 0.3424 | 14 | 95047762 | 11693 | 112 |
| *C8A* | 0.41126 | 0.34304 | 1 | 57320469 | 63427 | 195 |
| *C9* | 0.66717 | 0.346 | 5 | 39284242 | 80355 | 207 |
| *AFP* | 0.30889 | 0.35498 | 4 | 74301937 | 19954 | 34 |
| *SERPINA4* | 0.20004 | 0.35605 | 14 | 95027778 | 8465 | 53 |
| *FGB* | 0.26478 | 0.358 | 4 | 155484131 | 9784 | 40 |
| *LBP* | 0.05757 | 0.36365 | 20 | 36974884 | 30771 | 119 |
| *HNF4A* | 0.46018 | 0.36891 | 20 | 43029895 | 23388 | 175 |
| *LECT2* | 0.72343 | 0.36989 | 5 | 135282592 | 8080 | 64 |
| *ITIH3* | 0.46727 | 0.37456 | 3 | 52828783 | 14242 | 25 |
| *PZP* | 0.51725 | 0.37695 | 12 | 9301435 | 59556 | 122 |
| *GNMT* | 0.47036 | 0.38272 | 6 | 42928491 | 3127 | 31 |
| *AGTR1* | 0.42532 | 0.38395 | 3 | 148447966 | 12824 | 111 |
| *SAA1* | 0.57278 | 0.38849 | 11 | 18287810 | 3704 | 38 |
| *SAA2* | 0.04169 | 0.3973 | 11 | 18266786 | 3429 | 55 |
| *SLC22A7* | 0.37945 | 0.39854 | 6 | 43265997 | 7279 | 14 |
| *F12* | 0.43851 | 0.40054 | 5 | 176829138 | 7439 | 9 |
| *GATM* | 0.57238 | 0.40064 | 15 | 45653321 | 17421 | 96 |
| *CYP2E1* | 0.89995 | 0.40089 | 10 | 135340866 | 11761 | 83 |
| *ITIH1* | 0.82347 | 0.4011 | 3 | 52813941 | 12137 | 38 |
| *KHK* | 0.42636 | 0.4068 | 2 | 27309639 | 13991 | 18 |
| *CYP2A6* | 0.59304 | 0.423 | 19 | 41349445 | 6907 | 30 |
| *SLC22A1* | 0.63336 | 0.42453 | 6 | 160542862 | 36888 | 71 |
| *COLEC10* | 0.78771 | 0.42512 | 8 | 120079485 | 41209 | 598 |
| *FABP1* | 0.33624 | 0.43352 | 2 | 88422507 | 5074 | 27 |
| *APOC1* | 0.83934 | 0.43464 | 19 | 45417864 | 4737 | 29 |
| *AADAC* | 0.43001 | 0.43468 | 3 | 151531903 | 14373 | 101 |
| *APOA1* | 0.44757 | 0.44799 | 11 | 116706466 | 1872 | 46 |
| *NR1I3* | 0.57989 | 0.45028 | 1 | 161199458 | 8535 | 36 |
| *RDH16* | 0.4476 | 0.45155 | 12 | 57345214 | 7944 | 29 |
| *ABCC2* | 0.32495 | 0.4519 | 10 | 101542396 | 69955 | 99 |
| *HAL* | 0.65067 | 0.4546 | 12 | 96366439 | 23683 | 90 |
| *AOX1* | 0.5554 | 0.45597 | 2 | 201450736 | 85481 | 210 |
| *SPP2* | 0.39463 | 0.45941 | 2 | 234959345 | 26433 | 123 |
| *SERPINA10* | 0.58996 | 0.46017 | 14 | 94749649 | 9712 | 33 |
| *SERPIND1* | 0.72654 | 0.46263 | 22 | 21128400 | 13608 | 51 |
| *APOC2* | 0.87919 | 0.46288 | 19 | 45449307 | 3515 | 61 |
| *HGD* | 0.3846 | 0.4659 | 3 | 120347018 | 54068 | 38 |
| *SERPINF2* | 0.66085 | 0.46863 | 17 | 1646319 | 12240 | 57 |
| *BHMT2* | 0.66786 | 0.47169 | 5 | 78365589 | 20303 | 55 |
| *PON1* | 0.38889 | 0.48316 | 7 | 94926987 | 26857 | 88 |
| *TM4SF4* | 0.30723 | 0.49027 | 3 | 149192483 | 28698 | 177 |
| *ARG1* | 0.62552 | 0.49163 | 6 | 131894365 | 11104 | 19 |
| *HP* | 0.1611 | 0.50233 | 16 | 72088490 | 6464 | 23 |
| *LIPC* | 0.4996 | 0.50266 | 15 | 58724189 | 137854 | 411 |
| *VTN* | 0.45691 | 0.51054 | 17 | 26694304 | 3024 | 15 |
| *AFM* | 0.70408 | 0.52341 | 4 | 74347461 | 22257 | 36 |
| *APOC3* | 0.78674 | 0.52633 | 11 | 116700622 | 3166 | 60 |
| *C4BPB* | 0.15664 | 0.52637 | 1 | 207262627 | 10710 | 30 |
| *GYS2* | 0.60099 | 0.52707 | 12 | 21689040 | 68741 | 165 |
| *NR0B2* | 0.5333 | 0.5333 | 1 | 27237979 | 2487 | 1 |
| *CYP3A43* | 0.69597 | 0.53749 | 7 | 99425635 | 38081 | 12 |
| *AKR1D1* | 0.44742 | 0.53907 | 7 | 137761204 | 41847 | 113 |
| *CYP2J2* | 0.56977 | 0.55699 | 1 | 60358979 | 33466 | 46 |
| *TFR2* | 0.3551 | 0.57031 | 7 | 100218042 | 13231 | 39 |
| *ADRA1A* | 0.68497 | 0.57408 | 8 | 26636569 | 86603 | 350 |
| *HMGCS2* | 0.60322 | 0.57422 | 1 | 120290625 | 20903 | 45 |
| *PROZ* | 0.74794 | 0.58022 | 13 | 113812967 | 13731 | 63 |
| *SLC25A18* | 0.41325 | 0.59307 | 22 | 18043197 | 30459 | 67 |
| *MASP2* | 0.66607 | 0.59411 | 1 | 11104854 | 2442 | 55 |
| *AZGP1* | 0.70397 | 0.60307 | 7 | 99564352 | 9302 | 20 |
| *AGT* | 0.76641 | 0.61255 | 1 | 230838269 | 11599 | 80 |
| *CYP2C8* | 0.73006 | 0.61349 | 10 | 96796528 | 32726 | 109 |
| *ITIH2* | 0.76776 | 0.61597 | 10 | 7745278 | 46205 | 125 |
| *GC* | 0.7689 | 0.6183 | 4 | 72607413 | 42383 | 149 |
| *C6* | 0.92591 | 0.62084 | 5 | 41142247 | 71387 | 240 |
| *CYP3A4* | 0.75307 | 0.62722 | 7 | 99354589 | 27218 | 16 |
| *ADH1B* | 0.86182 | 0.62982 | 4 | 100226127 | 16431 | 36 |
| *G6PC* | 0.61795 | 0.63172 | 17 | 41052815 | 13640 | 11 |
| *PLG* | 0.79714 | 0.63635 | 6 | 161123224 | 9680 | 67 |
| *ADH4* | 0.90986 | 0.64527 | 4 | 100044807 | 20642 | 27 |
| *CYP2C9* | 0.91815 | 0.64539 | 10 | 96698414 | 51434 | 77 |
| *HPN* | 0.6094 | 0.65526 | 19 | 35531409 | 26069 | 75 |
| *HPD* | 0.78799 | 0.65565 | 12 | 122277432 | 19333 | 107 |
| *PROC* | 0.70474 | 0.65834 | 2 | 128175995 | 10827 | 43 |
| *SLC13A5* | 0.87692 | 0.66106 | 17 | 6588038 | 28650 | 102 |
| *AKR1C4* | 0.92083 | 0.66609 | 10 | 5238799 | 22113 | 126 |
| *SLC38A4* | 0.84947 | 0.67029 | 12 | 47158543 | 61237 | 179 |
| *APOC4* | 0.98016 | 0.67612 | 19 | 45445493 | 3260 | 42 |
| *UPB1* | 0.71238 | 0.69148 | 22 | 24891299 | 33059 | 60 |
| *UGT2B4* | 0.8842 | 0.69237 | 4 | 70345882 | 15741 | 193 |
| *AHSG* | 0.92365 | 0.69259 | 3 | 186330848 | 8259 | 40 |
| *A1BG* | 0.63004 | 0.69526 | 19 | 58856548 | 8310 | 28 |
| *C8G* | 0.8433 | 0.72 | 9 | 139839694 | 1733 | 11 |
| *APCS* | 0.67886 | 0.72952 | 1 | 159557615 | 1040 | 16 |
| *NR1I2* | 0.95784 | 0.736 | 3 | 119501556 | 35776 | 71 |
| *SAA4* | 0.85936 | 0.73688 | 11 | 18252901 | 5448 | 19 |
| *AGXT* | 0.838 | 0.74546 | 2 | 241808240 | 11677 | 107 |
| *PKLR* | 0.76838 | 0.74889 | 1 | 155259083 | 11660 | 27 |
| *CYP2C18* | 0.88664 | 0.75696 | 10 | 96443485 | 52462 | 154 |
| *SDS* | 0.8707 | 0.76164 | 12 | 113830249 | 11443 | 25 |
| *TMPRSS6* | 0.80417 | 0.77237 | 22 | 37461475 | 38218 | 120 |
| *SLC27A5* | 0.78005 | 0.77309 | 19 | 59009699 | 13660 | 41 |
| *BHMT* | 0.84954 | 0.80238 | 5 | 78407631 | 20480 | 60 |
| *HPX* | 0.81577 | 0.80494 | 11 | 6452278 | 9939 | 30 |
| *DMGDH* | 0.85959 | 0.80748 | 5 | 78316489 | 49008 | 229 |
| *F13B* | 0.87397 | 0.81347 | 1 | 197008320 | 28077 | 42 |
| *ADH6* | 0.78558 | 0.83721 | 4 | 100123795 | 16608 | 28 |
| *SLC10A1* | 0.9158 | 0.86005 | 14 | 70242132 | 21826 | 33 |
| *CYP2D6* | 0.9069 | 0.86595 | 22 | 42522500 | 4312 | 37 |
| *KLKB1* | 0.9958 | 0.87862 | 4 | 187148660 | 30965 | 143 |
| *F11* | 0.88447 | 0.87951 | 4 | 187187342 | 8709 | 66 |
| *CRP* | 0.818 | 0.88073 | 1 | 159682078 | 2301 | 21 |
| *ADH1A* | 0.86557 | 0.88665 | 4 | 100197525 | 14617 | 29 |
| *OIT3* | 0.97951 | 0.92023 | 10 | 74653496 | 39298 | 31 |
| *APOB* | 0.87955 | 0.92739 | 2 | 21224300 | 42645 | 43 |
| *ABAT* | 0.92321 | 0.92914 | 16 | 8814569 | 63857 | 397 |
| *ANGPTL3* | 0.8272 | 0.94318 | 1 | 63063190 | 8794 | 12 |
| *BAAT* | 0.9308 | 0.94514 | 9 | 104122698 | 23103 | 33 |
| *SLC2A2* | 0.88784 | 0.94544 | 3 | 170714135 | 30374 | 19 |
| *ORM2* | 0.85296 | 0.95604 | 9 | 117092068 | 3468 | 38 |
| *MAT1A* | 0.91827 | 0.96475 | 10 | 82031575 | 17839 | 19 |
| *GLYAT* | 0.982 | 0.97867 | 11 | 58477560 | 21856 | 76 |
| *SERPINA6* | 0.99779 | 0.98314 | 14 | 94770584 | 19089 | 65 |
| *CPS1* | 0.96379 | 0.9889 | 2 | 211458078 | 85753 | 381 |
| *PCSK6* | 0.94844 | 0.99261 | 15 | 101923928 | 105982 | 752 |

Note. CHR: chromosome; BP: base pairs.

**Supplementary Table 2.** Association *p*-values of genes frequently intergraded by HBV

| Gene Symbol | GATES-*P* | ECS-*P* | CHR | Start Position | Length (BP) | Number of SNPs |
| --- | --- | --- | --- | --- | --- | --- |
| *ITPR1* | 0.10145 | 0.39545 | 3 | 4535031 | 354493 | 748 |
| *ESRRG* | 0.12806 | 0.26442 | 1 | 216676592 | 220179 | 1562 |
| *TERT* | 0.1373 | 0.3183 | 5 | 1253286 | 41897 | 36 |
| *ITPR2* | 0.1537 | 0.86347 | 12 | 26488284 | 497843 | 953 |
| *SENP5* | 0.19217 | 1 | 3 | 196594790 | 66794 | 119 |
| *NTRK2* | 0.27777 | 0.60967 | 9 | 87320815 | 109802 | 691 |
| *MCM8* | 0.42406 | 0.10314 | 20 | 5931297 | 44555 | 106 |
| *IRAK2* | 0.47158 | 0.29605 | 3 | 10206602 | 78827 | 178 |
| *LRP1B* | 0.57285 | 0.40699 | 2 | 140988991 | 1899594 | 4634 |
| *PLXNA4* | 0.61336 | 0.25029 | 7 | 132169520 | 91709 | 819 |
| *ARHGEF12* | 0.61752 | 0.5909 | 11 | 120256035 | 104611 | 167 |
| *PHACTR4* | 0.63263 | 0.52987 | 1 | 28764660 | 62216 | 227 |
| *MYOM1* | 0.63737 | 0.20229 | 18 | 3066804 | 153162 | 358 |
| *ROCK1* | 0.64239 | 0.5551 | 18 | 18526866 | 164908 | 77 |
| *CTDSPL2* | 0.65338 | 0.7305 | 15 | 44719826 | 101410 | 169 |
| *FN1* | 0.7141 | 0.51859 | 2 | 216279383 | 21408 | 65 |
| *CYP2C8* | 0.73006 | 0.61349 | 10 | 96796528 | 32726 | 109 |
| *SMAD5* | 0.74349 | 0.69763 | 5 | 135468533 | 49888 | 114 |
| *MAPK1* | 0.80462 | 0.48982 | 22 | 22123318 | 98652 | 175 |
| *RAI1* | 0.8176 | 0.34002 | 17 | 17584771 | 129994 | 104 |
| *ADH1B* | 0.86182 | 0.62982 | 4 | 100226127 | 16431 | 36 |
| *CCNE1* | 0.87394 | 0.71059 | 19 | 30302897 | 12322 | 59 |
| *THRAP3* | 0.91704 | 0.84582 | 1 | 36690043 | 80914 | 27 |
| *RBFOX1* | 0.94394 | 0.09568 | 16 | 7382845 | 376938 | 7581 |
| *CPS1* | 0.96379 | 0.9889 | 2 | 211458078 | 85753 | 381 |
| *LRFN2* | 0.96765 | 0.98504 | 6 | 40359329 | 195774 | 583 |

Note. CHR: chromosome; BP: base pairs.

**Supplementary Table 3.** Association *p*-values of genes with significant association in previous studies

| Gene Symbol | GATES-*P* | ECS-*P* | CHR | Start Position | Length (BP) | Number of SNPs |
| --- | --- | --- | --- | --- | --- | --- |
| *GRIK1* | 0.11605 | 0.02725 | 21 | 30971114 | 341200 | 855 |
| *HLA-DQB1* | 0.1888 | 0.12695 | 6 | 32627244 | 7190 | 60 |
| *EFCAB11* | 0.22645 | 0.20823 | 14 | 90389620 | 31260 | 282 |
| *GRIN2A* | 0.34964 | 0.7291 | 16 | 9847264 | 428660 | 1028 |
| *C2* | 0.35786 | 0.31106 | 6 | 31895493 | 7342 | 55 |
| *HLA-DPB1* | 0.47615 | 0.67542 | 6 | 33043766 | 13707 | 304 |
| *HLA-DPA1* | 0.56332 | 0.15205 | 6 | 33032345 | 9109 | 451 |
| *GLB1* | 0.63817 | 0.59177 | 3 | 33038108 | 100216 | 304 |

Note. CHR: chromosome; BP: base pairs.

**Supplementary Table 4.** Association *p*-values of COSMIC HCC risk genes

| Gene Symbol | GATES-*P* | ECS-*P* | CHR | Start Position | Length (BP) | Number of SNPs |
| --- | --- | --- | --- | --- | --- | --- |
| *PTEN* | 0.12295 | 0.0501 | 10 | 89623381 | 108306 | 48 |
| *TERT* | 0.1373 | 0.3183 | 5 | 1253286 | 41897 | 36 |
| *PTPRB* | 0.17326 | 0.20978 | 12 | 70909649 | 94155 | 236 |
| *HNF1A* | 0.21946 | 0.07092 | 12 | 121416370 | 23944 | 80 |
| *PIK3CA* | 0.24972 | 0.08035 | 3 | 178866144 | 91737 | 123 |
| *RB1* | 0.33316 | 0.36565 | 13 | 48877882 | 178144 | 162 |
| *KMT2D* | 0.38902 | 0.32115 | 12 | 49412757 | 36350 | 21 |
| *ATM* | 0.42108 | 0.34572 | 11 | 108094255 | 5836 | 141 |
| *TP53* | 0.49947 | 0.32245 | 17 | 7571719 | 6843 | 49 |
| *CREBBP* | 0.55471 | 0.85066 | 16 | 3775055 | 155066 | 123 |
| *ARID1A* | 0.70739 | 0.70739 | 1 | 27022505 | 86090 | 4 |
| *IL6ST* | 0.77223 | 0.74711 | 5 | 55230924 | 59867 | 16 |
| *TSC2* | 0.80018 | 0.9022 | 16 | 2098202 | 40519 | 12 |
| *NFE2L2* | 0.82243 | 0.91774 | 2 | 178095036 | 33823 | 51 |
| *ARID2* | 0.86833 | 0.87352 | 12 | 46123491 | 164208 | 91 |
| *CTNNB1* | 0.9115 | 0.83207 | 3 | 41240995 | 40939 | 41 |
| *AXIN1* | 0.93463 | 0.84148 | 16 | 337439 | 65284 | 191 |
| *ATR* | 0.94529 | 0.84685 | 3 | 142168076 | 129499 | 138 |
| *KMT2C* | 0.99774 | 0.9886 | 7 | 151832009 | 301079 | 240 |
| *CDKN2A* | 0.9992 | 0.95765 | 9 | 21967750 | 7076 | 39 |

Note. CHR: chromosome; BP: base pairs.
